# A Charge Swap mutation E461K in the yeast dynamin Vps1 reduces endocytic invagination

**DOI:** 10.1080/19420889.2015.1051274

**Published:** 2015-08-31

**Authors:** Sarah E Palmer, Iwona I Smaczynska-de Rooij, Christopher J Marklew, Ellen G Allwood, Ritu Mishra, Martin W Goldberg, Kathryn R Ayscough

**Affiliations:** 1 Department of Biomedical Science; University of Sheffield; Sheffield, UK; 2 Department of Biological Science; Durham University; Durham, UK

**Keywords:** dynamin, dynamin-like protein, endocytosis, membrane trafficking

## Abstract

Vps1 is the yeast dynamin-like protein that functions during several membrane trafficking events including traffic from Golgi to vacuole, endosomal recycling and endocytosis. Vps1 can also function in peroxisomal fission indicating that its ability to drive membrane fission is relatively promiscuous. It has been of interest therefore that several mutations have been identified in Vps1 that only disrupt its endocytic function. Most recently, disruption of the interaction with actin through mutation of residues in one of the central stalk α helices (RR457,458 EE) has been shown to disrupt endocytosis and cause an accumulation of highly elongated invaginations in cells. This data supports the idea that an interaction between Vps1 and actin is important to drive the scission stage in endocytosis. Another Vps1 mutant generated in the study was vps1 E461K. Here we show data demonstrating that the E461K mutation also disrupts endocytosis but at an early stage, resulting in inhibition of the invagination step itself.

Dynamins are a family of large GTPases that function in membrane scission events. Dynamin-1 is the best characterized of these proteins and its function as a scission factor during endocytosis is well studied. While dynamin1 is expressed in cells of neuronal origin dynamin 2 is expressed in most tissue types. Furthermore, while dynamin-2 functions during endocytosis, more recent evidence has emerged for functions at other stages of membrane trafficking giving rise to the possibility of a more general fission function, similar to Vps1.^1^ Vps1 is the only dynamin-like protein functioning in membrane trafficking processes in yeast. While its function in endocytosis has previously been controversial this is likely due to some redundancy in its fission function with the amphiphysins (Rvs161/Rvs167) and also because GFP tagging of the protein is likely to significantly impair its ability to incorporate into its oligomeric ring structures and localize to endocytic sites. Reports from a number of labs have shown defects in endocytosis in cells lacking *vps1*; interactions with endocytic proteins; and localization to endocytic sites in cells co-expressing untagged and tagged protein.^2-6^ In addition, mutations in Vps1 affecting endocytic but not other functions of the protein, are difficult to reconcile with the proposal that the protein only functions indirectly in endocytosis.^7,8^

An interaction between actin and mammalian dynamin was mapped to a specific helix in the stalk domain of the protein.^9^ Mutations in this region indicated that the actin interaction is important for stress fiber formation in podocytes but not for endocytosis. However given that these studies were performed in HeLa cells where actin is not required for endocytosis, any defect on transferrin uptake caused by the mutation may have been subtle. We generated equivalent charge swap mutations in yeast Vps1 to determine whether an interaction with actin was conserved and if so, whether it was important for any or all functions of the protein.^10^ Vps1 was shown to bind actin and the vps1 RR-EE mutation reduced actin binding as predicted. A detailed analysis of the scission defects caused by this mutation are described in our recent paper.^10^ One of the other mutations, vps1 E461K (**Fig. 1A**), showed similar actin binding to wild type Vps1 in a high-speed pelleting assay (**Fig. 1B**). Further analysis revealed that vps1 E461K is able to form oligomeric ring structures similar in size to the wild type protein (30.2 nm SD 4.9 nm n = 44; compared to 32 nm SD 3.7 n = 49 for wild type).

**Figure 1.**
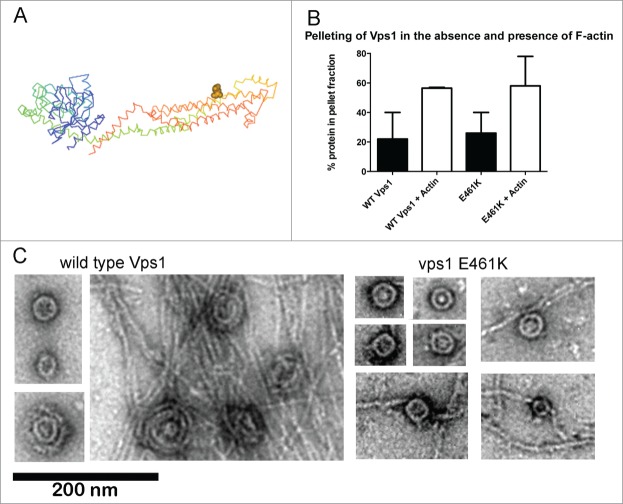
In vitro analyses of vps1 E461K. (**A**) Structure of Dynamin-like protein- (pdb protein 4BEJ) with the residue equivalent to E461K (E426) highlighted on one of the helices of the dynamin stalk domain. (**B**) Actin binding of Vps1 wild type and the E461K mutant was assessed by incubating with F-actin followed by high speed centrifugation. The proportion of Vps1 found in the pellet in the presence and absence of actin is shown. Error bars are standard deviation. The proportion of actin in the pellet at high speed is not significantly different. (**C**) Wild type and mutant Vps1 were purified and analyzed using transmission electron microscopy following negative staining. For wild type, images shown are single and double rings in the absence of actin, and the double rings that form in the presence of actin filaments. For the E461K mutant images of 4 single rings are shown in the absence of actin and then rings in the presence of actin. No double rings were observed.

**Figure 2.**
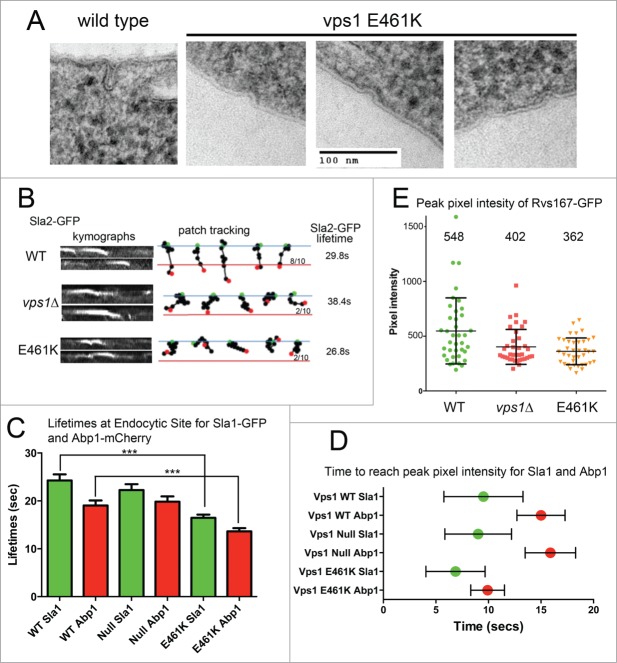
Analysis of cells expressing vps1 E461K. (**A**) Cells expressing wild type or vps1 E461K were high pressure frozen as previously described. Thin sections were imaged to observe plasma membrane invaginations. Examples of representative invaginations are shown. (**B**) Cells expressing wild type or vps1 E461K in the presence of the endocytic reporter Sla2-GFP were analyzed in live cells. Movies were recorded of endocytic patch movement and used to generate kymographs shown in the left panel. Patches were tracked and the ability of the patch to track further than 200 nm was recorded. The blue line denotes the plasma membrane while the red line is 200 nm into the cell from this point. The green spot indicates starting point, and the red spot the point of disassembly or where the patch moved away from the focal plane. Ten patches were analyzed for each and the proportion crossing the line noted. The lifetime of ≥30 patches for each strain was analyzed and noted on the right. (**C**) Endocytic patches detected with endocytic reporters Sla1-GFP and Abp1-mCherry were analyzed in wild type or *vps1 E461K* cells. Movies of cells were recorded and used to measure lifetimes of the reporters in 30 patches. Shown is lifetime ±SEM. P value <0 .0001 (***) from Students t tests indicates lifetimes for both Sla1 and Abp1 are significantly shorter that corresponding wild type values. (**D**) To determine whether E461K affects recruitment times at the endocytic site Sla1-GFP and Abp1-mCherry were analyzed in co-expression studies in live cells. At 30 individual sites the time taken for each marker to reach peak intensity was recorded. The green or red spot marks the mean time, and the error bar denotes range. (**E**) The peak pixel intensity of ≥37 patches of Rvs167-GFP were analyzed in wild type, vps1 null or vps1 E461K expressing cells. Numbers indicated are the mean intensity. E461K intensity is lower than that in wild type cells (p = 0.002 in students' t-test).

However while 55% of rings observed in the presence of actin oligomerized further to a double ring structure, no double rings were observed for vps1 E461K protein indicating that actin could not induce higher order Vps1 structures in this mutant protein. In addition, and possibly coupled to the absence of double ring structures, actin bundling was not induced by the Vps1 E461K mutant (**Fig. 1C**).

When expressed as the sole vps1 protein in cells the *vps1 E461K* was able to rescue the majority of defects associated with the *vps1* deletion, including temperature sensitivity, trafficking of carboxypeptidase Y to the vacuole, endosomal recycling and peroxisome fission.^10^ However analysis of fluid phase uptake indicated a delay in endocytosis. Using electron microscopy of cells fixed by high pressure freezing, a high proportion of short/shallow invaginations were observed with a mean length of 18 ± 7nm n = 50 invaginations, compared to 50–60 nm for invagination length in wild type cells. Live cell imaging analysis revealed reduced levels of successful invagination of the reporter Sla2-GFP (**Fig. 2B**). The lifetime of the Sla2-GFP patches was also shorter, indicating premature disassembly of complexes at the site. Endocytic site assembly was also investigated using dual reporters: Sla1-GFP to assess early stage site assembly and Abp1-mCherry to determine effects at the level of actin assembly. As with Sla2, both reporters showed reduced lifetime at the endocytic site in the Vps1 E461K expressing cells (**Fig. 2C**). Analysis of the behavior of 30 patches, and the point at which peak fluorescence intensity at a site was observed for both Sla1 and Abp1, revealed that Abp1-mCherry appeared to be recruited more rapidly at sites in the presence of E461K supporting the idea that dynamins have a function at endocytic sites prior to scission (**Fig. 2D**). However, following this Abp1recruitment, the site disassembled quickly, corresponding to the lack of invagination also indicated by the electron microscopy and invagination patch tracks (**Fig. 2A and B**). Finally, analysis of the peak pixel intensity of the amphiphysin involved in scission (Rvs167-GFP) revealed that reduced levels of Rvs167 were observed at endocytic sites (**Fig. 2E**). These levels were significantly less than those at patches in wild type cells but not significantly different from the levels of Rvs167 at patches in *vps1* null cells. A reduced level of Rvs167 suggests a defect early in invagination rather than at the later scission stage when, as observed in the wild type situation or in the vps1 RR-EE mutant, higher levels of Rvs167 are observed.^10^

Taken together the data indicate that a form of *vps1* carrying a mutation E461K in the proposed actin binding helix of the central stalk domain is still able to bind actin. However, actin binding does not lead to increased oligomerization of Vps1 as observed with wild-type protein. We hypothesize that the inhibition of further oligomerization precludes appropriate membrane tubulation so that observed invaginations are less deep than in wild type cells and that proteins disassemble from the endocytic site prematurely. Thus, we propose that Vps1, like dynamin, might function in a feedback loop during endocytosis with actin facilitating oligomerization of Vps1 (defective in vps1 E461K) which then leads to an increased capacity for actin bundling (defective in vps1 RR-EE), culminating in a successful scission and release of an endocytic vesicle.^10^
